# Eating in the upside down: a critical look at the new U.S. dietary guidelines from a mediterranean perspective

**DOI:** 10.1007/s40519-026-01828-6

**Published:** 2026-02-22

**Authors:** Daniele Del Rio

**Affiliations:** 1 Human Nutrition Unit, Department of Food and Drug, University of Parma, Parma, Italy; 2 OnFoods Foundation, Parma, Italy

On 7 January 2026, the U.S. Department of Health and Human Services (HHS) and the Department of Agriculture (USDA) released the new edition of the *Dietary Guidelines for Americans* (DGA) 2025–2030, presenting them as a “reset” of federal nutrition policy with a simple slogan: “eat real food.” [1].

These guidelines are far from a technical document for insiders. In the United States, they orient public food programmes, such as school and community catering, as well as health communication campaigns, with a potentially enormous impact on everyday diets. For that reason, it is worth reading them carefully, separating what is sensible from what risks becoming a communicative and public-health boomerang.

## The diet of Americans could certainly be better

In response to the updated DGA, the Italian Society of Human Nutrition (SINU) issued a position statement recalling a key fact: in the U.S., a very high proportion of daily energy intake comes from highly processed foods with high energy density [2]. These products are estimated to account for around 60% of total energy intake, whereas in Italy the figure is much lower (around 20%).

In this context, the appeal to “real food”, fresh foods prepared and cooked simply, is neither trivial nor purely rhetorical. It represents an attempt to reverse a cultural trend linked to obesity and to the risk of conditions such as diabetes and cardiovascular diseases.

The new DGA indeed call for a “dramatic reduction” in highly processed foods, rich in refined grains, added sugars, salt, “unhealthy fats” (a definition that readers should keep in mind, as I will refer to it later in this piece), and additives. This recommendation is reasonable and important from a health perspective, because it refers primarily to the nutritional composition of certain commercial products that should clearly be consumed in moderation.

I find much more problematic the way the DGA embrace the concept of “ultra-processed foods,” which risks demonising, in a way unsupported by science, the technological processes used to produce commercial foods. In the vast majority of cases, those processes are designed to ensure food safety and wholesomeness and, crucially, to extend shelf-life, reducing both waste and health risks. For those who wish to delve into the issue, debate around the definition and meaning of “ultra-processed food” is lively on social media and in the nutrition research community. My own position is laid out in several works [see 3 and 4, for example]. In brief, the available evidence on associations between different ultra-processed foods and health outcomes, as well as the studies examining specific food additives, raises serious doubts that processing per se is the main causal factor. It is quite possible that other, unmeasured confounders play a substantial role. For this reason, blanket recommendations to limit or avoid all foods labelled as “ultra-processed” currently rest on a weak scientific foundation and should be considered fragile. In parallel, rapid public-policy interventions framed around this concept, such as those hinted at in the DGA, seem premature and should be reconsidered carefully before implementation.

## Added sugars: perhaps a step forward?

On added sugars, SINU’s position converges with that of the Department of Nutrition at Harvard University [5], in recognising that the new edition of the DGA is more explicit than previous versions. The guidelines state that no amount of added sugars (or sweeteners) is “recommended”, and they offer a simple operational rule: *no more than 10 g of added sugars per meal*.

Often, guidelines refer generically to “simple sugars,” thereby lumping together those naturally present in foods and those added during processing or at the table. Yet sugars naturally present in fruit cannot be equated with the sugars added to a soft drink, for instance. This distinction is important, even if, from a consumer’s perspective, it remains difficult to appreciate in practice.

However, if we consider the recommended daily portions of fruit and dairy (milk and/or yoghurt), very little room is left before reaching the well-known limit of 10% of energy from simple sugars. In other words, even in current guidelines, there is effectively no space left for added sugars.

## The “protein hype”: more protein for everyone, but on what basis?

One of the most debated features of the new DGA is the increase in recommended protein intake for adults to 1.2–1.6 g/kg/day—substantially higher than the values recommended by most countries and by international health bodies.

SINU’s critique focuses on two main issues. First, there is a lack of clarity regarding protein sources. It is not sufficient to say “more protein” without distinguishing between fish, legumes, cheese, eggs, red meat, processed meat, and so on. The health implications of these sources are very different, and this nuance is only marginally addressed. Second, the methodological basis for the new recommendation appears weak. According to the background documentation, the higher protein range is largely extrapolated from studies conducted with weight loss as the primary outcome, with follow-up periods too short to evaluate other endpoints, such as cardiometabolic risk in people who are not overweight.

The Harvard's T.H. Chan School of Public Health echoes these concerns [5], warning that increasing protein in general, without guiding the public towards better sources, could have unintended consequences. Many people already consume sufficient protein, while the real discriminant is often the quality of the protein-containing foods. The problem is not to demonise protein (nor animal protein per se), but to avoid turning the message “more protein” into a free pass to increase red meat and cheese intake, precisely where the literature tends to favour plant proteins and fish.

## An upside down pyramid and animal fats: a confusing picture

Another controversial element is the image used to launch the guidelines: a food pyramid that is literally inverted compared to the traditional one, familiar to most consumers, often associated with the Mediterranean diet. Leaving aside the desire to please the many fans of *Stranger Things* and its “Upside Down” world, this image is at odds with some aspects of the guidelines themselves, particularly in relation to fat recommendations. The DGA reaffirm the globally recognised limit of no more than 10% of daily calories from saturated fats. Yet, in the pyramid, foods and condiments rich in saturated fats like red meat, whole milk, cheese, butter, and even beef tallow as a cooking fat, are prominently displayed.

SINU has taken a clear position on this point, stressing that the re-branding of animal fats as “healthy fats” is not supported by strong evidence, especially given their impact on blood lipid and cholesterol profiles. This stance is perfectly aligned with major international guidelines, which continue to recommend limiting saturated fats in favour of unsaturated fats, predominantly of plant origin. The Harvard's T.H. Chan School of Public Health [5] adds a practical argument: simple calculations suggest that if one chooses full-fat dairy to meet daily portions and uses butter or tallow for cooking, it becomes very easy to exceed the 10% energy limit from saturated fats set by the DGA themselves, even before considering the rest of the diet. For an average citizen, not specialised in nutrition, striking images and simple slogans are more likely to be retained than numerical limits. It is entirely plausible that people will remember the repeated “real food” message and the attractive images of “butter and steak” placed high in the pyramid, while the 10% saturated fat limit will remain in the small print, requiring much closer reading.

This is a poor communication strategy, compounded by a lack of clarity about what the pyramid’s structure actually represents. Is it meant to reflect recommended consumption frequency, or simply to display the various food groups? The confusion extends to legumes, which are scarcely highlighted, and to whole grains, which paradoxically end up at the very top of the inverted pyramid, despite being recommended in the text at 2–4 portions per day.

## The great absentees: environmental sustainability and socioeconomic context

SINU and colleagues at the Harvard's T.H. Chan School of Public Health also agree on another major gap: the new DGA do not explicitly take into account either the environmental impact or the socioeconomic dimension of their recommendations. Today, anyone drafting dietary guidelines for a population should be asking questions such as: *Who has access to these foods? What are the implications for supply chains, prices, and ecosystems? Are these recommendations compatible with a One Health perspective, which integrates human, animal, and environmental health?* Ignoring these aspects means that the recommendations will be effectively unattainable for a substantial share of the population. Moreover, it risks encouraging production and consumption patterns with negative environmental effects that, in the long term, may prove even more serious than the direct impacts on consumer health.

## Conclusions: what message reaches an Italian (or European) consumer?

For an Italian consumer, the message “more fruit and vegetables, more attention to labels and added sugars” found in the new U.S. guidelines is certainly acceptable and consistent with the best available evidence, but it is also exactly what our own national guidelines have been saying for many years.

On the message “fewer ultra-processed foods, more simple foods,” I have already expressed my reservations at length, particularly regarding the scientific robustness of the “ultra-processed” construct as a policy tool.

What should be forgotten, or even firmly rejected, in my view, are the indistinct emphasis on higher protein intakes, the rehabilitation of animal fats, and the confusing graphics of the inverted pyramid, devoid of a clear legend that would make it immediately interpretable. Rather than this “Upside Down” pyramid, we would do better to hold on to the strong cultural and scientific reference of the Mediterranean diet, with its inherent links to sustainability, conviviality and culinary tradition. We should also remember the Food Pyramid recently developed by SINU, which synthesises the most up-to-date scientific evidence on the relationship between diet and human health and explicitly incorporates sustainability dimensions.

In short, just like the pyramid that represents them, these new U.S. dietary guidelines are, in many respects, “upside down.” And if I may borrow once more from *Stranger Things*, I would rather leave the Upside Down where it belongs, a fictional dark, decaying, parallel dimension, than adopt it as a model for public health nutrition.

## Data Availability

No datasets were generated or analysed during the current study.
